# Correlation between Histological Status of the Pulp and Its Response to Sensibility Tests

**DOI:** 10.22037/iej.2017.04

**Published:** 2017

**Authors:** Mandana Naseri, Akbar Khayat, Sara Zamaheni, Shiva Shojaeian

**Affiliations:** a *Department of Endodontics, Dental School, Shahid Beheshti University of Medical Sciences, Tehran, Iran; *; b *Shiraz University Dental School, Shiraz, Iran; *; c *Department of Endodontic, University of British Columbia, Vancouver, Canada*

**Keywords:** Clinical Diagnosis, Histologic, Pulp Tests

## Abstract

**Introduction:**

The purpose of this study was to assess the accuracy of sensibility tests by correlating it with histologic pulp condition.

**Methods and Materials:**

Assessment of clinical signs and symptoms were performed on 65 permanent teeth that were scheduled to be extracted for periodontal, prosthodontic or orthodontic reasons. The normal pulp and reversible pulpitis were considered as treatable tooth conditions while irreversible pulpitis and necrosis were considered as untreatable conditions. The teeth were then extracted and sectioned for histological analysis of dental pulp. Histologic status and classification corresponded to the treatable or untreatable pulp condition. Comparisons between histological treatable and untreatable pulp condition were performed with chi-square analysis for sensibility test responses. The positive predictive value (PPV), negative predictive value (NPV) and accuracy to detect untreatable from treatable pulp condition were calculated for each test.

**Results:**

A significant difference was detected in the normal and a sharp lingered response to heat and cold tests. There was significant difference in the negative response to EPT between histological groups. The kappa agreement coefficient between clinical and histological diagnosis of pulp condition was about 0.843 (*P*<0.001). The accuracy of cold and heat tests and EPT to detect treatable pulp or untreatable pulp states were 78, 74 and 62%, respectively. The sensibility tests diagnosed untreatable pulpitis with a higher probability (NPV=63%-67% -54%, PPV=83%-91% -95% for heat, cold and EPT, respectively).

**Conclusion:**

Sensibility test results were more likely to diagnose pulpal disease or untreatable pulp conditions. However, to increase the diagnostic accuracy patient history, clinical signs and symptoms and also radiographic findings in conjunction with sensibility tests must be used. The result of this small study demonstrated a good agreement between clinical and histological pulp diagnosis.

## Introduction

Cariogenic bacteria are the most common causes of pulpitis. However, the degree of pulp inflammation cannot be determined based on the size of carious lesion [1, 2]. Histological reactions within the pulp including various immunity and inflammatory responses are triggered in dental pulp and may even occur due to initial caries limited to enamel and according to caries extension [3]. 

Accurate diagnosis of pulp conditions is critical for proper treatment planning. The clinicians, based on their clinical diagnosis, decide on selection of a conservative restorative treatment, vital pulp therapy or root canal therapy. Vitality or necrosis of dental pulp and more importantly reversible or irreversible nature of pulpitis are determined based on patient’s clinical signs and symptoms, sensibility tests including thermal and electrical pulp tests (EPT) and radiographic assessment; however clinical diagnosis is challenging when results of sensibility test are inconsistent with subjective findings [4]. A review of the literature shows that a few studies have assessed the accuracy of clinical methods in diagnosis of pulp conditions [5]. Although it has been demonstrated that using a combination of diagnostic tests improves accurate diagnosis, sufficient evidence is lacking on the accuracy of diagnostic pulp tests or clinical signs and symptoms to identify pulp conditions [5, 6]. With recent advances in vital pulp therapy for treatment of pulpitis and preservation of pulp vitality, it is extremely important to accurately diagnose the pulpal conditions and know how well the clinical diagnosis correlates with the actual pulp state[7].

The purpose of this clinical/histological study was to assess the accuracy of sensibility tests and the correlation between the results of sensibility tests and histologic pulp condition.

## Materials and Methods

This study was approved in the Ethics Committee of Shahid Beheshti University, Tehran, Iran. Sixty-five permanent incisors and premolars that were scheduled for extraction due to periodontal disease, prosthodontic or orthodontic purposes, *etc.* at Oral and Maxillofacial Surgery of Dental School of Shahid Beheshti Medical University, Iran, were evaluated in this study. All teeth had mature apices and were collected from patients aged between 20 to 55 years old. Clinical data were collected in predesigned questionnaires, including patient̕ s information, tooth number, medical and dental history, characteristics of pain, intra- and extra-oral examinations, sensibility tests and clinical diagnosis of pulp disease. The patients were excluded if they received analgesic drugs before the examination.

All the clinical examinations were accomplished by a single examiner. Heat and cold tests were performed using a hot gutta-percha stick (GC Corporation, Tokyo, Japan) and an Endo-ice (Hygenic Corp., Akron, OH, USA) sprayed onto a cotton pellet, respectively. Two teeth were tested as controls before the experimental one to observe a baseline normal response. All the teeth were isolated with cotton rolls and dried thoroughly before testing. The testing site was confined to the middle of the buccal surface for 10 sec. In case of no response after one min, the tests were repeated at the cervical, and occlusal thirds to ensure no response to the stimuli. Severity, quality and duration of pulpal response to sensibility tests were recorded as normal or uncomfortable pain, sharp or dull and short or lingering, respectively.

Electrical pulp testing (EPT) was carried out on both, experimental and control teeth using an electric pulp tester (Parkell, Edgewood, NY, USA) according to the manufacturer’s instructions. Toothpaste was the conducting medium and the probe was placed on intact tooth structure [7]. A response before an 80 reading was recorded as positive. The diagnosis of pulp diseases was made based on the following classification [8]: normal pulp had no symptom and showed normal responses to clinical testing. The pulp responded to sensibility tests as mild transient sensation that did not cause patient distress. Reversible pulpitis produced a rapid and sharp response. The stimulation was uncomfortable to the patient but quickly reversed after removal the irritation. There were no significant radiographic changes in the periapical region of the suspected tooth and the experienced pain was not spontaneous. Irreversible pulpitis produced a sharp or dull pain that continued after the elimination of stimulant and caused lingered pain. The pain might be spontaneous or referral and awakened patients during the night. Necrotic pulp gives no response to sensibility tests. The normal pulp and reversible pulpitis were considered as treatable conditions that lead to conservative treatment if needed while the irreversible pulpitis and pulp necrosis were considered as untreatable condition which required root canal therapy and extirpation of the pulp. 

***Histological processing***

After performing clinical examinations, the teeth were extracted gently, under local anesthesia. Immediately after extraction, the apical one-third of the roots were cut by a high speed handpiece and #169 plain tapered carbide fissure bur (SWS, Switzerland) under water spray for better penetration of fixative according to Seltzer and Dummer [9, 10].

The teeth were immersed in 10% formalin solution at 4^°^C for 3 days. Next, the specimens were stored in 10% formic acid for 12 days for hard tissue demineralization. The solution was stirred 3 times a day and exchanged daily. In order to ensure demineralization, radiography was taken and compared with the control radiograph of the intact tooth. The teeth were then rinsed under running water, dehydrated in ascending grades of ethanol, cleared in xylene (Merck, Darmstadt, Germany) and double embedded in paraffin blocks. Paraffin blocks were then longitudinally and serially sectioned into 8 μm thick slices. Of each 3 sections, one was chosen and stained with Hematoxylin and Eosin for histological analysis. To assess the penetration depth of bacteria into dentin, some of the specimens were Gram stained. 

At least two calibrated investigators evaluated the specimens under light microscope. Pulp reaction to stimuli was evaluated in different areas of the coronal and radicular pulp tissue to locate the area with the most severe inflammatory reaction and contained the carious lesion. Changes in the odontoblastic layer, vascular alterations, type and extent of inflammatory cells, presence of abscess, necrosis or calcification, and internal resorption were all evaluated in histopathological sections and the type of pulp disease were determined according to Seltzer’s classification [10]:

***Intact un-inflamed pulp (IUP):*** No significant changes are seen in cells and pulp vessels and the odontoblastic layer is intact. 

***Hyperemic pulpitis (HP):*** Dilated blood vessels and blood congestion are apparent. There is some slight changes in the odontoblastic layer of some specimens but no sign of inflammatory cells exists. 

***Transient pulpitis (TP):*** Chronic inflammatory cells, mainly lymphocytes, macrophages and plasma cells, are scattered in the pulp tissue and more commonly in the coronal pulp, but inflammatory exudate has yet to be formed. 

***Retrogressive pulp (RP): ***The pulp tissue is atrophic, number and size of pulp cells such as odontoblasts and fibroblasts have decreased. Number of collagen fibers has been visibly increased and deposition of reparative dentin can be seen in some areas. Chronic inflammatory cells are not prominent in this group.

***Chronic partial pulpitis (CPP):*** Chronic inflammatory exudate of the pulp is locally seen. Inflammation is limited to a small area in the coronal section and has not yet extended to the radicular pulp. Irregular arrangement of odontoblasts and disseminated or local vacuolization are seen. Congestion of local blood vessels is evident. In most specimens, calcification is seen in different forms. In some specimens, small abscesses are seen in the pulp beneath the carious lesion with necrosis. 

***Chronic total pulpitis (CTP):*** The inflammation is similar to the previous group but with greater extent of involvement. The entire pulp chamber and radicular pulp are also involved. Coronally, an area of liquefaction or coagulation necrosis was always discerned. The remainder of the pulp contained granulation tissue. 

***Partial necrosis (PN)***: No vital or cellular tissue is seen in the coronal section adjacent to the carious lesion. Severe inflammation and abundant infiltration of inflammatory cells are seen in radicular pulp. Bacteria have direct access to pulpal space. 

***Total necrosis (TN):*** In the entire pulp, no sign of odontoblasts is seen. Blood vessels have been completely destructed and inflammatory cells are being disintegrated. 

Although different pathological conditions may be simultaneously observed in dental pulp tissue, one specific state is often dominant; which was considered as the final diagnosis. In Seltzer’s classification, IUP, HP, TP, AP, CPP (without necrosis) correspond to treatable pulp condition and CPP (with necrosis), CTP , PN, TN correspond to untreatable pulp condition [11].

Statistical comparisons between histological treatable and untreatable pulp condition were performed with chi-square analysis for sensibility test responses and Kappa agreement between histological and clinical diagnosis was calculated. The positive predictive value (PPV) (True Positive/True Positive+False Positive), negative predictive value (NPV) (True Negative/True Negative+False Negative) and accuracy (AC) (True Positive+True Negative/Total) to detect untreatable from treatable pulp condition were calculated for each test.

## Results

**Figure 1:**
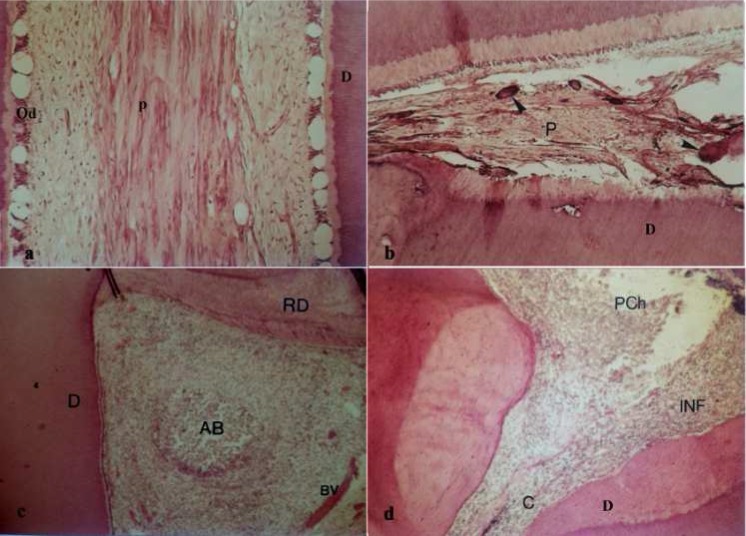
Different histological patterns of the pulp in teeth with similar response to sensibility tests. *A)* Transient pulpitis and *B*) Retrogressive pulp in teeth response normal to termal test. [D, Dentin. P, Pulp. Od, Odontoblast layer. (magnification 100×)]; *C)* Chronic total pulpitis and *D*) Partial necrosis in teeth response normal to cold test. [AB, Abscess. RD, Reparative Dentin. C, root canal. Pch, Pulp chamber. INF, Inflamation. BV, Blood Vessle (magnification 100×)]

Table 1 shows the distribution of histological pulp conditions as well as their correlation with the type of pulp responses to sensibility tests. None of the sensibility test results was correlated with a specific histological pattern of dental pulp. Different histological patterns of the pulp were seen in teeth that gave similar responses to tests (Figure 1). 

Chi-square test; with Bonferroni correction, detected a significant difference in the normal (*P*<0.001) and a sharp lingered (*P*=0.04) response to heat test between two histological treatable and untreatable groups.

A significant difference was detected in normal (*P*<0.001) and sharp linger (*P*=0.004) responses to cold test with a marginally significant difference for no responses (*P*=0.064) between two histological treatable and untreatable groups. There was significant difference in the negative response to EPT between treatable and untreatable histological groups but the positive response to EPT was not significantly different between two histological groups.

The AC, PPV, and NPV for thermal tests and EPT results to detect treatable or untreatable pulp conditions were calculated and are summarized in Table 2.

The kappa agreement coefficient between clinical and histological diagnosis of pulp condition was about 0.843 which was significant (*P*<0.001). A significant difference were detected in normal, irreversible and necrosis (*P*<0.001) diagnosis between two histological groups (Table 3).

## Discussion

In the current study, we investigated the correlation of pulp response to sensibility tests and histologic pulp condition. Analysis of thermal test results revealed that the lingering sharp or no response to cold test was significantly correlated with untreatable pulpal conditions, including irreversible pulpitis and necrosis. However, short sharp pulpal response to cold test, which is a sign of reversible pulpitis, was not significantly related to a histological diagnosis as in several cases, the pulpitis was deemed untreatable. Cold test had a higher likelihood to diagnose pulpal disease or untreatable pulp conditions (PPV=91%>NPV=67%). 

Lingering sharp response to heat test had a significant correlation with untreatable pulp condition. But, short sharp or no response did not have a significant correlation with histological diagnosis. Thus, the heat test also diagnosed untreatable pulpitis with a higher probability (PPV=83%, NPV=63%). No response to EPT indicated untreatable pulpal condition but positive response to EPT could not differentiate treatable from untreatable pulpitis.

**Table 1:** The correlation of pulp responses to sensibility tests with histological pulp conditions

**Histological diagnosis**	**Cold**					**Heat**					**EPT**		**N (%)**
	**Normal**	**Sharp pain**		**Dull**	**-**	**Normal**	**Sharp pain**		**Dull**	**-**	**+**	**-**	
		**Short **	**Lingering**				**Short**	**Lingering**					
**Normal**	5	1	-	-	1	5	1	-	-	1	7	-	7 (10%)
**Hyperemia**	2	1	-	-		1	1	-	-	1	3	-	3 (4.6%)
**Transitional**	5	1	-	-	1	5	1			1	6	1	7 (10.8)
**Retrogressive**	2	-	-	-	-	2	-	-	-	-	2	--	2 (3.1%)
**CPP without necrosis**	2	3	-	-	1	2	2		-	2	6	-	6 (9.2%)
**CPP with necrosis**	1	-	4	-	-	1		3	-	1	5	-	5 (7.7%)
**CTP**	-	4	1	-	1	1	2	2	-	1	6	-	6 (9.2%)
**Partial necrosis**	-	3	3	-	4	-	3	4	-	3	6	4	10 (15.4%)
**Total necrosis**	1	2	5	-	11	1	3	3	-	12	3	16	19 (29%)
**Treatable**	16	6	-	-	3	15	4	1	-	5	24	1	25 (38.5%
**Untreatable**	2	9	13	-	16	2	9	12	-	17	20	20	40 (61.5%)

Previous studies have evaluated the predictive value and accuracy of sensibility tests for identifying pulp vitality or necrosis. A cold test has often shown accuracy over 90% [12-14]. The accuracy of a heat test and EPT was reported 86% and 76%, respectively [13, 14]. But, this study analyzed sensibility test results to differentiate between the treatable and untreatable pulp which help clinician to make a decision for selection of a conservative treatment or extirpation of pulp tissue and root canal treatment. Hyman *et al. *[15], in a retrospective study showed that diagnostic tests were much poorer as positive predictors of disease than as negative predictors and there have been cases with normal histological condition that were diagnosed as pulpitis by diagnostic tests. Cisneros-Cabello [16] found a significant but low correlation between painful response to thermal stimuli and histological pattern of dental pulp and concluded that pain on thermal stimuli, particularly cold, should not be used as a reliable reference for diagnosis and treatment planning.

Thermal tests followed by EPT are the most commonly used techniques for the assessment of pulp conditions [17]. However, these tests, known as the sensibility tests, only assess the response of nerve fibers in dental pulp and since the nervous tissue is highly resistant to inflammation, the nerve fibers may still remain active after the degeneration of pulp tissue. Therefore, these tests cannot accurately indicate pulp vitality or presence of adequate blood supply [18]. Subjectivity and difficult interpretation are another drawbacks of sensibility pulp tests. Yet, a more precise interpretation may be possibly obtained *via* a comparison with the response of a control tooth [17]. Basically, the clinician make a clinical diagnosis of pulp condition based on patient’s clinical signs and symptoms, pulp sensibility tests and radiographic examination. However, no accurate method has been described for definite diagnosis of pulp conditions [5]. Accuracy of diagnostic tests indicates the level of agreement between the test results and the reference test and Predictive Value is often calculated for diagnostic tests to assess the likelihood of a correct diagnosis. PPV is the probability that a positive test result actually represents a disease-positive person. NPV is the probability that a person with a negative test result is actually free of disease. However, this value depends on the prevalence of disease and changes with the prevalence rate [15, 19].

**Table 2.** The accuracy, positive predictive value (PPV), and negative predictive value (NPV) for thermal tests and EPT

	**Sensibility tests (%)**		
	**Cold**	**Heat**	**EPT**
**PPV**	91	83	95
**NPV**	67	63	54
**Accuracy**	78	74	62

This study demonstrated a good agreement between clinical and histological pulp diagnoses. However, this correlation is not too accurate. In particular, no significant association was found between the clinical and histological diagnoses of reversible pulpitis. Some cases of clinical diagnosis of reversible pulpitis existed that were histologically diagnosed as untreatable pulpitis. Dental pulps were histologically analyzed as the reference test to assess the real pulp condition. We used human teeth scheduled for extraction due to different purposes, so having all pulpal conditions from normal to necrotic ones to reduce a risk of spectrum bias [20]. Teeth serial sections were made in such way that they all contained the carious lesion. This allowed assessment of the entire pulp tissue related to the carious site. Yet, preparation of specimens, histological interpretation of pulp status and distinction of different pulp conditions are all challenging. The other limitation of this study was that it was not possible to diagnose asymptomatic irreversible pulp condition clinically because we were not ethically allowed to perform clinical procedures including caries removal. Also, radiographic findings were not used to diagnose pulp condition. Asymptomatic irreversible cases usually have no clinical symptoms and respond normally to sensibility tests but deep caries would result in exposure following caries removal.

In recent years, small number of studies have assessed the accuracy of clinical diagnosis and their correlation with the histological pulp status. Some studies have found no clear correlation between clinical symptoms and pulp responses to sensibility tests with the histological pattern of dental pulp [9, 10, 21, 22]. Other studies have found partial correlation between clinical sign and symptom with histological findings [16, 23]. A recent study demonstrated a good agreement between clinical and histologic diagnosis of pulp condition, especially for cases with normal pulp and reversible pulpitis [24]. Our results are somewhat consistent with their observation. However, this study found significant correlation between clinical and histologic diagnosis of normal pulp, irreversible pulpitis and also necrotic pulp. This inconsistency may be due to the different histologic criteria. Histologic pulp classification used in both studies has been related to early 70s [10]. It seems necessary to reevaluate histologic pulp classification according to the new knowledge in pulp healing potential and regeneration in future studies.

In conclusion, sensibility tests had a higher likelihood to diagnose pulpal disease or untreatable pulp conditions. To increase the diagnostic accuracy, patient history, clinical signs and symptoms and also radiographic findings in conjunction with sensibility tests must be used. Recently, using biological markers of inflammation has also been suggested for the assessment of pulp conditions in terms of reversibility or irreversibility of inflammation [25-27]. However, the diagnostic value of this method has yet to be identified. Confirming a reliable standard objective and non-invasive method for accurate assessment of pulp conditions needs further investigation in future studies.

**Table 3:** The correlation of clinical and histological diagnosis of pulp condition

**Diagnosis**	**Normal **	**Reversible pulpitis**	**Irreversible pulpitis**	**Necrosis**	**Number of teeth**
**Treatable **	1	7	0	-	25
**Untreatable**	2	3	20	15	40
**Total**	20	10	20	15	65
